# Effect of Proteins on the Network Formation and Degradation of Peroxide Cross-Linked Natural Rubber Elucidated by Time-Domain NMR

**DOI:** 10.3390/polym17081063

**Published:** 2025-04-15

**Authors:** Adun Nimpaiboon, Antonio González-Jiménez, Roberto Pérez-Aparicio, Fernando Martín-Salamanca, Zenen Zepeda-Rodríguez, Juan López-Valentín, Jitladda Sakdapipanich

**Affiliations:** 1 Rubber Technology Research Centre (RTEC), Faculty of Science, Mahidol University, Nakhon Pathom 73170, Thailand; adun.nim@mahidol.ac.th; 2 Department of Chemistry and Center of Excellence for Innovation in Chemistry, Faculty of Science, Mahidol University, Bangkok 10400, Thailand; 3 Plastic Research Center (AIMPLAS), València Parc Tecnològic, C/Gustave Eiffel 4, 46980 Paterna, Spain; angonzalez@aimplas.es; 4 Escuela Superior de Ingeniería y Tecnología (ESIT), Universidad Internacional de La Rioja (UNIR), Av. de la Paz 137, 26006 Logroño, Spain; 5 Institute of Polymer Science and Technology (CSIC), C/Juan de la Cierva 3, 2800 Madrid, Spain; rperezaparicio@gmail.com (R.P.-A.); fms@ictp.csic.es (F.M.-S.); zenen@ictp.csic.es (Z.Z.-R.); 6 Department of Chemistry and Center of Excellence for Innovation in Chemistry, Faculty of Science, Mahidol University, Nakhon Pathom 73170, Thailand

**Keywords:** biopolymers, natural rubber, proteins, sustainability, multiple-quantum NMR

## Abstract

The importance of sustainable polymers has increased greatly in the last years since most polymers are derived from non-renewable sources. Sustainable polymers (i.e., biopolymers) such as natural rubber (NR) are proposed as a solution for this concern. A comparative study between NR and deproteinized NR (DPNR) was carried out to elucidate the role of proteins on the network formation and degradation of peroxide cross-linked NR using time-domain NMR experiments. The ^1^H multiple-quantum (MQ) NMR experiments provided information on the cross-link density and its spatial distribution, while the actual fraction of non-coupled network defects was obtained by exploiting the Hahn echo approach measured on swollen samples. The results showed that proteins influenced the network formation during the vulcanization process of NR, leading to a higher number of non-elastic network defects and promoting the creation of additional cross-links with a broader spatial distribution. The formation of network heterogeneities in different length scales deeply influences the mechanical properties of NR samples. On the other hand, the proteins showed a pro-oxidant activity on the degradation behavior by accelerating the degradation process of peroxide cross-linked NR.

## 1. Introduction

Sustainable rubbers are natural materials or materials derived from renewable, recycled, and waste carbon resources and their combinations, which at the end of life can be recycled, biodegraded, or composted [1,2,3]. Sustainable rubbers also exhibit a reduced environmental impact throughout their life cycle. Biopolymers can be derived from renewable resources, or they can be partially made from renewables and synthesized like traditional polymers, as in the case of bio-based polymers [4]. Rubber from *Hevea brasiliensis*, known as natural rubber (NR), has been recognized as the most important biopolymer in the rubber industry, although more than 2000 species of plants and fungi have also been found to produce rubber hydrocarbon. NR is a very high-molecular-weight and long-chain branched biopolymer, composed primarily of *cis*-1,4-polyisoprene containing about 6% of non-rubber constituents such as proteins, lipids, carbohydrates, and other ingredients [5]. NR is a unique material for many industrial as well as medical applications, such as aircraft and truck tires, surgical gloves, and condoms, owing to its superior crack growth resistance and excellent tensile properties in comparison with other naturally occurring and synthetic polyisoprenes. The structural characterization of NR using high-resolution ^13^C and ^1^H NMR revealed that the fundamental structure of NR is long-chain isoprene units with end-groups, i.e., with initiating ω-terminal groups and terminating α-terminal groups. The ω-terminal group is not revealed yet but has been presumed to interact with proteins [6,7]. The α-terminal group is proposed as mono- or di-phosphate linked with phospholipids [8,9]. These findings suggest that the interaction of proteins and lipids at the molecular chain ends of NR possibly forms a three-dimensional pseudo-end-linked network, contributing to the numerous characteristics of un-vulcanized NR, such as gel formation [7,10] or strain-induced crystallization [11]. Therefore, proteins are one of the essential components believed to bring about these marvelous properties of NR. However, some proteins exuding from NR products are found to be associated with the severe allergic reactions termed a *Type I* allergy, which is potentially serious or even life-threatening. The U. S. Food and Drug Administration (FDA) issued a medical alert to rubber device producers in 1991 after a number of patients died of this protein allergy [12]. Proteins in latex can usually be removed by the enzymatic decomposition of proteins in the presence of surfactant, followed by centrifugation [11,13,14].

The cross-linking process of rubber is generally known as vulcanization, where three-dimensional networks are formed by chemical or physical methods [15,16]. The most useful vulcanizing systems in rubber technology are sulfur/accelerator systems and organic peroxides [17,18,19,20,21]. The role of proteins on cross-linked NR was studied based on accelerated sulfur systems. It was proven that the proteins and nitrogenous compounds in NR act as natural accelerators for sulfur vulcanization [22], as evidenced by the deproteinized NR (DPNR) that demonstrates a slower cure as well as a lower cross-link density compared to raw NR. In addition, the proteins play a strong role in the network structure and distribution of the three types of sulfidic cross-linking [22]. Peroxide vulcanization is preferred in some applications due to the good heat resistance, low compression set, and high transparency of NR products. Moreover, the absence of some additives offers a solution for solving the nitrosamine contamination as a carcinogen and *Type IV* allergy (allergic contact dermatitis) caused by the amine-based accelerators in sulfur vulcanization [23]. As the peroxide vulcanization process takes place via a radical mechanism, the proteins in NR that might associate with radicals need to be clarified.

Several techniques such as inverse gas chromatography [24], osmometry [25], mechanical analysis [26], equilibrium swelling experiments [27], dielectric measurements [28], high-resolution solid-state NMR [29,30], or scattering techniques [31,32] have been used to partially elucidate the network structure of elastomers. Recently, time-domain solid-state NMR has been shown to be a robust and versatile technique to obtain direct and local evidence about the different parameters that define the polymer network structure [33,34,35,36,37,38,39]. This is based on the direct measurement of residual dipolar couplings that persist because of the presence of cross-links and other topological constraints, such as entanglements, that restrict the number of accessible segmental conformations, which leads to local chain ordering caused by the non-isotropic fast segmental motions. Many NMR experiments, e.g., ^1^H transverse relaxation [40], two-dimensional (2D) magnetization exchange spectroscopy [41], and Hahn, together with solid echoes [42], were used to determine various parameters controlled by residual dipolar couplings. Nevertheless, it is already well known that ^1^H double-quantum (DQ) (or more generally multiple-quantum, MQ) NMR can be considered the most quantitative and reliable method for the measurement of weak residual dipolar couplings [37,43]. By suitable data analysis, the coherent dipolar effect, related to the number of constraints and their spatial distribution (inhomogeneities), could be independently separated from the temperature-dependent segmental dynamics for rubber networks [37].

The aim of this work is to study the role of proteins in the network formation and degradation process of NR vulcanized with an organic peroxide system by means of different time-domain NMR experiments performed in low-field spectrometers. In this sense, the ^1^H MQ-NMR experiment was used to evaluate the evolution of cross-link density and the spatial distribution of cross-links for NR and DPNR samples during the peroxide vulcanization as well as during the degradation process. Additionally, the actual fraction of non-coupled network defects, e.g., free chain ends, dangling chain ends, or loops, was obtained by applying Hahn echo experiments on swollen samples with deuterated solvent. The contributions of those proteins to the network structure and its influence on the network formation in NR is crucial to determine quantitative knowledge about the most important biopolymer in the rubber industry.

## 2. Experimentation

### 2.1. Sample Preparation

NR was prepared by diluting fresh NR (FNR) latex to 30% dry rubber content (DRC) and adding 1% (*w*/*v*) formic acid to coagulate the latex. The coagulum was rinsed with water several times and then dried in an oven at 70 °C for 24 h.

DPNR was carried out by incubating 30% DRC of the FNR latex with 0.04% (*w*/*v*) proteolytic enzyme (KP-3939, Kao Co., Tokyo, Japan) and 1% (*w*/*v*) sodium dodecyl sulfate (SDS) at 37 °C for 12 h, followed by centrifugation twice. The cream phase was cast on a glass plate and dried under the same method as the NR.

The NR and DPNR were compounded with 1 part per hundred rubber (phr) of dicumyl peroxide (DCP) in an open two-roll mill using the standard procedure and then vulcanized by compression molding at 160 °C by varying times.

### 2.2. Characterization

The protein content was measured using nitrogen analysis using a LECO Nitrogen Analyzer (model FP 528) (LECO, St. Joseph, MO, USA) and generally calculated using a universal conversion factor of 6.25.

The content of the long-chain fatty acid ester group was determined by FTIR measurements based upon a calibration using a series of mixtures of methyl stearate and synthetic *cis*-1,4-polyisoprene (Kuraprene IR10). The quantity of fatty acid ester groups per weight of rubber was determined by the intensity ratio of the bands at 1739 cm^−1^ (C=O) to 1664 cm^−1^ (C=C).

The quantitative analysis of the metal content was determined by inductively coupled plasma atomic emission spectroscopy (ICP-AES).

The cure behavior and the optimum cure time (t_95_) were monitored by the Rubber Process Analyzer (RPA) (Alpha Technologies^®^ RPA 2000^™^, Heilbronn, Germany), where t_95_ is the time required for the torque of the rheometer to increase up to 95% of the total torque at 160 °C.

The time-domain proton NMR experiments were performed on a Bruker minispec mq20 spectrometer (Bruker, Billerica, MA, USA) operating at a resonance frequency of 20 MHz, with a 90° pulse length of 3 μs and a dead time of 12 μs. The sample temperature was controlled with an air-operated BVT3000 heater (Bruker, Billerica, MA, USA).

The tensile tests were performed on type 2 dumbbell specimens according to ISO 37 in an Instron model 3365 dynamometer (Instron, Norwood, AR, USA) equipped with a video camera to measure deformations at a strain rate of 500 mm/min.

#### 2.2.1. ^1^H DQ-NMR Experiment

Time-domain ^1^H DQ-NMR spectroscopy is based on the measurement of the residual dipolar couplings (NMR observable) between protons originating from the orientational anisotropy due to constraints, such as cross-links and entanglements. Such local anisotropy can be described by a non-zero dynamical orientation of the polymer backbone $\langle P_{2}(\cos\theta )\rangle$ (second-order Legendre polynomial). The NMR experiments are in the timescale related to the length *R* and orientation α of the end-to-end vector. Assuming a freely jointed rigid rod chain model, the average segment orientation is as follows:

(1) $$\langle P_{2}(\cos\theta )\rangle \approx \frac{3}{5}\frac{R^{2}}{N^{2}b^{2}}\frac{3\cos^{2}\alpha -1}{2}\approx S_{b}\frac{3\cos^{2}\alpha -1}{2}$$

where *N* is the number of statistical segments between constraints and *b* is the statistical segment length. The overall NMR signal is the contribution of the individual monomer or segment signals, which are at least partially time-averaged over the distribution of the end-to-end vectors between constraints. From this, an average value of the local backbone orientation, $S_{b}$, with respect to the end-to-end vector, arises (Equation (1)). For ideal chains *R*^2^ ≈ *Nb*^2^, it may be written as follows:

(2) $$S_{b}\approx \frac{3}{5}\frac{R^{2}}{N^{2}b^{2}}\propto \frac{1}{N}\approx \upsilon$$

being proportional to the constraint density *υ*. Since the proton dipolar coupling depends on molecular orientation, *S_b_* is detected in NMR because it gives a non-zero residual dipolar coupling and, then, is calculated from the experimental average residual dipolar coupling constant *D_res_*, by comparison with its static counterpart, *D_stat_*, as follows (*k* is a correction factor <1 accounting for the spin arrangement and motions within a statistical segment):

(3) $$S_{b}=k\frac{D_{res}}{D_{stat}}$$

According to Equations (2) and (3), *D_res_* is inversely proportional to the average molecular weight of the rubber chains between constraints *M_x_*. There are different models [44] that describe the contribution of cross-links (*M_c_*) and entanglements (*M_e_*) to the NMR observable (*D_res_*) and the measured order parameter (*S_b_*). Assuming the additivity of entanglement and cross-link constraints [45,46], we may write *D_res_* ∝ 1/*M_c_ +* 1/*M_e_*.

The ^1^H DQ-NMR experiment technique is based on enhanced Baum–Pines pulse sequences [37,47]. The raw experimental data from such experiments need to be normalized in such a way that the temperature-independent network structure effect (at temperatures far above the *T*_g_) can be separated from the temperature-dependent segmental dynamics without invoking any specific model, as was widely explained elsewhere [37,43,47]. A proper analysis of the NMR signals by means of a numerical inversion procedure based on the fast Tikhonov regularization analysis [48] provides the actual distribution of dipolar couplings that can be defined by the average apparent coupling constant *D_res_*, and the standard deviation, *σ*, characterizing the distribution width of the residual dipolar couplings.

From published spin dynamic simulations and assuming a reasonable model for the intra-segmental motions, the relationship between the experimental residual dipolar coupling constant, *D_res_*, and the molecular weight between constraints, *M_x_*, was obtained in the NR samples as the following equation [37]:

(4) $$M_{x}^{\left(NR\right)}=\frac{617\;Hz}{D_{res}/2\pi }[kg/mol]$$

assuming a constant entanglement density (then, $M_{x}^{\left(NR\right)}\propto \;M_{c}^{\left(NR\right)}$), tetra-functional cross-links, and a phantom network model (to take into account the non-affinity of real systems at the scale of the cross-links) [49], the cross-link density, $\upsilon_{NMR}$, can be determined by $\upsilon_{NMR}=1/M_{c}$.

#### 2.2.2. Hahn Echo Experiments Measured on Swollen Samples

The fraction of non-coupled network defects has been investigated by ^1^H DQ-NMR and Hahn echo experiments [37,49]. In the dry state, the fraction of non-coupled defects depends on temperature, and it is always underestimated because chain packing or topological constraints promote some restrictions in the fast-segmental motions of dangling chain segments and loops [43]. Thus, some fractions of network defects are, in fact, observed as dipolar coupled and elastically active segments because the slow arm retraction process is not able to relax the dangling chains in the experimental NMR timescale [50,51]. The topological restrictions are released, and the chain dynamics are speeded up by swelling the rubber networks with a suitable solvent, leading to fast isotropic motions. Consequently, the NMR experiments on the swollen samples provide a reliable measure of the actual fraction of non-coupled and elastically inactive network defects [49]. In the present work, the rubber samples were swollen in deuterated toluene at room temperature for 24 h in sealed dark vials in order to prevent them from degradation [27].

The complex thermodynamics and heterogeneous nature of the swelling process [27,52] hide substantial structural information from the stretched network, making nonsensical the use of complex DQ-NMR experiments to perform a deeper analysis of a direct signal that codifies the dipolar coupling information. For that reason, simpler Hahn echo experiments (which also have a dipolar origin for the transverse relaxation) were performed to identify and quantify the actual fraction of non-coupled polymer segments in the swollen samples.

## 3. Results and Discussion

### 3.1. Characterization of Rubber Samples

The characteristics of the rubber samples are given in Table 1. The NR samples contained 2.62% (*w*/*w*) of proteins that could be selectively removed by deproteinization, as evidenced by the very low protein content of DPNR. The ester content of the NR and DPNR, which represented the lipid part, showed similar values. The metal content (Cu^2+^, Fe^2+^, Mn^2+^) of both the NR and DPNR, which strongly influences the radical mechanism [53], was negligible.

### 3.2. Pseudo-End-Linked Network

Although the main goal of this work is to investigate the network structure formed by peroxide vulcanization, un-vulcanized NR also contains a pseudo-end-linked network that has been believed to bring about superior elastic properties rather than synthetic polyisoprene (IR) [54]. The cross-linking point of the end-linked network is classified into two types, i.e., (i) the intermolecular interaction of the proteins via hydrogen bonding at the initiating ω-terminal group [6,7] and (ii) the micelle formation or association of the polar head-groups of phospholipid molecules at the terminating α-terminal group [8,9]. Thus, the deproteinization process decomposes the network formed by the proteins in the DPNR, wherein the network derived from the phospholipids still remains. Additional trans-esterification treatment (explained in the Supplementary Information) removes the phospholipids and avoids the formation of the end-linked network. The proposed pseudo-end-linked network structure of NR, including the structural change after deproteinization and trans-esterification, can be seen in the Supplementary Information, Figure S1 [10].

**Figure 1:**
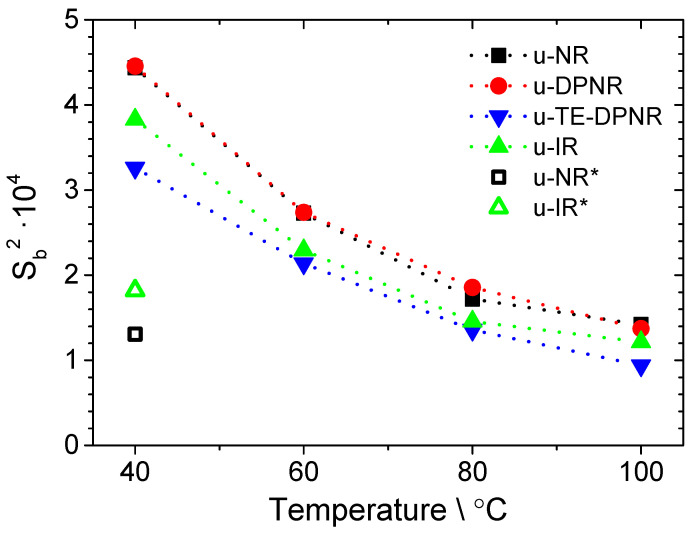
Temperature dependence of the squared local backbone orientation parameter ($S_{b}^{2}$) for un-vulcanized linear melt samples: natural rubber (u-NR), deproteinized natural rubber (u-DPNR), trans-esterified natural rubber DPNR (u-TE-DPNR), and synthetic polyisoprene (u-IR), respectively. On the other hand, u-NR* and u-IR* are samples subjected to mastication treatment on a two-roll mill for 15 min. The preparation details of the u-TE-DPNR and u-IR samples are explained in the Supplementary Information.

In the un-vulcanized NR samples, the existence of the pseudo-end-linked network has been proved to cause an apparent increment in molecular weight and the related development of entanglements and their dependent properties [55]. In this sense, it is well known that *D_res_* is sensitive and directly proportional to any constraints (independently of its nature) that restrict the segmental motion of polymer chains [56,57,58]. Therefore, ^1^H DQ-NMR experiments have been successfully used to analyze the segmental autocorrelation function of un-vulcanized (linear melt) samples [59,60], which is dominated by the restrictions imposed by entanglements and topological constraints (caused by the polymer molecular weight and chain architecture). Note that *D_res_* for a linear melt is an apparent quantity that depends on temperature and time. According to these statements, the variation in the average *D_res_* for the different un-vulcanized samples studied in this work (see Figure 1) must be related to changes in the topological constraints and chain mobility.

Figure 1 shows the temperature dependence of the squared local backbone orientation parameter, $S_{b}^{2}$, which corresponds to the orientation autocorrelation function at the timescale of the DQ-NMR experiments for the un-vulcanized samples: u-NR, u-DPNR, trans esterified DPNR (u-TE-DPNR), and synthetic polyisoprene (u-IR). $S_{b}^{2}$ decreases in the chosen temperature range and the actual NMR experimental time window is related to changes in segmental conformations inside the constrained space caused by faster dynamics in the reputation regime [61], where the dimensions of the apparent tube are determined by the real molecular weight between entanglements, *M_e_*, for each sample. In the case of u-NR and u-DPNR, they present higher $S_{b}^{2}$ values than the trans-esterified counterpart (u-TE-DPNR) at any temperature, which might reflect the presence of the pseudo-end-linked network that can promote higher anisotropy in the rubber motions. In this sense, the importance of phospholipids (absent in the u-TE-DPNR sample) on the formation of thermo-sensitive cross-link points should be more evident in comparison with the almost negligible effect that the proteins (u-DPNR) imposed on the chain motions of the u-NR sample. A synthetic u-IR sample was also investigated in order to compare it with the u-TE-DPNR sample. The differences between u-IR and u-TE-DPNR should be related to changes in the molecular weight. These results are completely aligned with the elastic properties of these samples: u-NR and u-DPNR showed almost the same properties, while u-TE-DPNR was quite similar to u-IR [54]. It can be confirmed that the topological constraints in NR should be dominated by the entanglements and the pseudo-end-linked network derived from phospholipids (with an almost negligible contribution from proteins). In this sense, the actual molecular weight between junction points was not properly estimated in the un-vulcanized samples because (i) this pseudo-end-linked network was unstable at temperatures above 100 °C [62] and (ii) at temperatures below 100 °C, the segmental dynamics in NR and DPNR were not fast enough to average out all the available conformations according to the restrictions caused by them.

It is worth pointing out that those samples were not subjected to any mechanical treatment, unlike the vulcanized samples, which were masticated during their compounding. In order to consider these effects on the rubber samples, Figure 1 also shows the $S_{b}^{2}$ value for the natural rubber (u-NR*) and synthetic polyisoprene (u-IR*) samples (measured at 40 °C) after a mastication treatment on a two-roll mill for 15 min (equivalent to the compounding step on the vulcanized samples). The results indicate a stronger effect of the mechanical treatment on the rubber chain molecular weight and, hence, on the entanglement effect in the case of the u-NR* compared to the synthetic counterpart. These results could be related to the effect of mastication on the pseudo-end-linked network in natural rubber.

**Figure 2:**
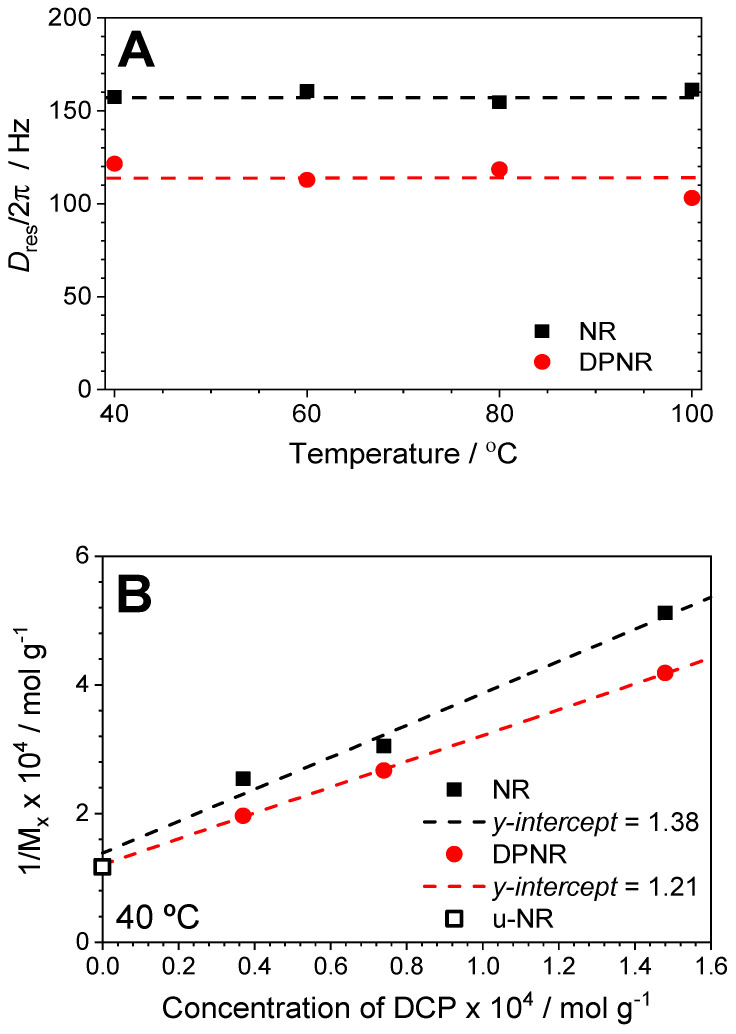
(**A**) Residual dipolar couplings, *D_res_*, for vulcanized NR and DPNR with 1 phr of DCP (3 min at 160 °C) as a function of the NMR measuring temperature. (**B**) Relationship between the density of constraints (given as 1/*M_x_* after 3 min of vulcanization time at 160 °C) and the concentration of DCP for the NR and DPNR samples measured at 40 °C. The y-intercept value represents the contribution of the entanglements (1/*M_e_*) to the total amount of constraints assuming additive contributions (1/*M_c_* + 1/*M_e_*). The result shown in (B) for the un-vulcanized sample subjected to mastication treatment on a two-roll mill for 15 min (u-NR*) reflects the apparent (time and temperature-dependent) restrictions obtained from the measured D_res_ for that linear rubber melt (see also Figure 1).

The temperature dependence of *D_res_* for the un-vulcanized rubber samples (see Figure 1) was completely suppressed in the vulcanized samples (3 min at 160 °C) in the temperature range from 40 °C to 120 °C for both the NR and DPNR samples, as shown in Figure 2A. This means that the measuring temperature is far above the rubber *T*_g_, and the segmental dynamics are fast enough to average out all the accessible conformations in the time window imposed by the NMR measurement [43]. Consequently, in those samples, *D_res_* reflects the constraints that define the rubber network structure without any interference from the polymer dynamics (temperature-dependent effects).

In order to demonstrate the linear relationship between the cross-link density measured by NMR and the concentration of peroxide for the NR and DPNR samples (see Figure 2B), additional samples containing 1, 2, and 4 phr of DCP were prepared under the same conditions, and vulcanized at 160 °C for 3 min, as expected. According to the previous explanation, the molecular weight between constraints, *M_x_*, measured by NMR, in fact, codifies the actual number of constraints imposed by (i) cross-links, (ii) entanglements (including those that are trapped), and (iii) the pseudo-end-linked network. Therefore, the extrapolated *y*-intercept value in Figure 2B represents the constraints in terms of *1*/*M_e_* for the NR and DPNR after the vulcanization process. The NR and DPNR show quite similar *y*-intercept values, which match with the *D_res_* value obtained in the un-vulcanized NR (u-NR*) sample after the mastication process (see Figure 1). These results point out the strong effect of mastication and vulcanization dominating over the pseudo-end-linked network structure formed by the proteins and phospholipids. The latter governs the outstanding properties of un-vulcanized NR (in terms of green strength) but has a negligible effect in the vulcanized samples, whose elastic properties mainly depends on the network structure formed by C-C cross-links (in the case of the peroxide-cured samples). Although the presence of proteins has a minimal effect on the topological constraints for the vulcanized samples (*y*-intercept), the slope in Figure 2B likely indicates a strong influence of these non-rubber substances on the vulcanization process, which undoubtedly enhanced the cross-linking efficiency in the NR, compared to the DPNR.

At this point, it is important to remark on some limitations by other experimental approaches to quantitatively analyze the effect of proteins on the actual network structure of NR in comparison with the ^1^H DQ-NMR experiments. On the one hand, the widely used swelling experiments provide only partial and qualitative information about the cross-link density because of the uncertainties associated with the complex thermodynamics of the swelling process and the elastic model that defines the behavior of swollen networks [27]. On the other hand, the most complete information about the NR network structure obtained by the small-angle neutron scattering (SANS) technique was intensely disturbed by the strong scattering of the protein aggregates [63].

Because of these statements, the evolution of the main parameters that define the network structure, e.g., the cross-link density and its spatial distribution, as well as the non-coupled network defects, during the vulcanization process have been deeply studied in the following sections by using ^1^H DQ-NMR experiments. These measurements were carried out at 40 °C because, at this temperature, the chain dynamics of the studied samples are fast enough to reach the plateau value of *D*_res_ (see Figure 2A). Additionally, this low temperature prevents the subsequent reactions of unreacted peroxides (especially at the initial steps of the vulcanization process) and NR degradation.

### 3.3. Evolution of Cross-Link Density During the Vulcanization Process

**Figure 3:**
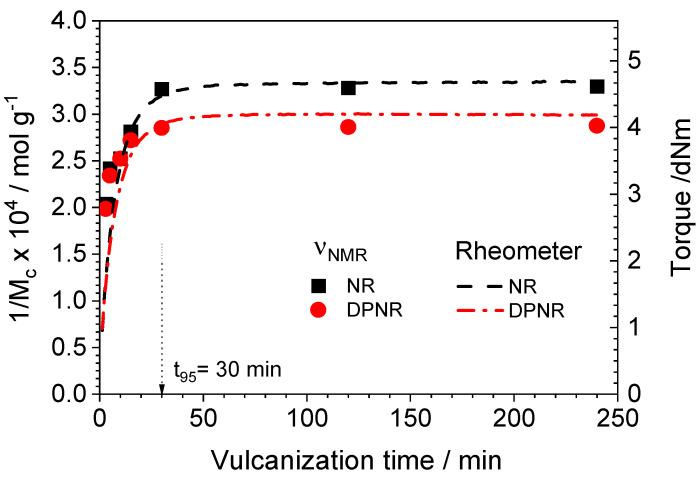
Evolution of cross-link formation for the NR and DPNR samples with 1 phr of DCP at 160 °C as a function of vulcanization time determined by NMR (symbols) and rheometer (dashed lines), respectively.

The formation of cross-links between polymer chains during the vulcanization process transforms rubber samples into an elastic material. It strongly influences their mechanical properties [64]. Figure 3 shows the evolution of cross-link formation for the NR and DPNR with the vulcanization time determined by NMR and rheometer measurements. A good correlation was clearly found between both techniques showing a characteristic curve for peroxide vulcanization. Peroxide vulcanization has a radical pathway that initiates the cross-linking reaction immediately after the homolytic scission of peroxide, as evidenced by the lack of scorch time in the vulcanization curve. The increased cross-link density (as measured by NMR) with the vulcanization time was reflected in the torque measured by the rheometer until a plateau was reached at the end of the process.

By comparing the NR and DPNR vulcanization curves, it is obvious that the proteins in NR increased the cross-link density without any influence on the vulcanization rate, since the optimum vulcanization time, defined as t_95_, is quite similar for both samples. In that sense, the proteins might act as a coagent, improving the efficiency of peroxide as a cross-linking agent. An enhancement in the efficiency of vulcanization via radical reaction could be associated with different mechanisms, such as the suppression of the non-network forming the side reactions, i.e., chain scission and disproportionation [65,66], as well as the formation of bridges between polymer chains contributing to additional cross-links [67]. The coagent bridges which are covalently bonded to the polymer chains are possibly a grafting of coagents [67,68], an interpenetrating network of homo-polymerized coagents [69], and an aggregation of coagents like filler particles [70,71], depending on several factors, such as the readiness of the coagent to homo-polymerize, the concentration polarity, and the solubility of the coagent [17,68]. The role of the existing protein in NR as a coagent was supported by the presence of radicals on the molecules of protein extracted from the NR. The electron paramagnetic resonance (ESR) technique (see Supplementary Information, Figure S2) can detect the presence of radicals on the proteins of NR, for which the *g* value at 2.004 corresponds to the carbon radicals on the backbone and the side chain of proteins similar to the results from the studies of amino acids and proteins examined elsewhere [72,73,74].

**Figure 4:**
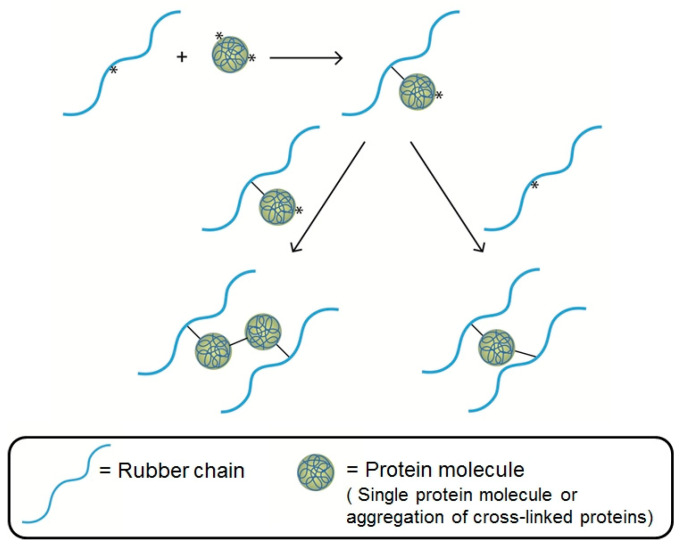
Schematic representation of the possible mechanism reaction of proteins as a coagent in peroxide cross-linked NR, where radicals have been represented by the symbol *. Adapted from ref. [75].

The proteins in NR are rather polar molecules, and therefore, their miscibility with the non-polar NR chains is quite poor. Such proteins tend to separate and then form aggregates of cross-linked proteins caused by the recombination of the protein radicals and homo-polymerized proteins at the unsaturated bond of the side chain, e.g., phenylalanine and tryptophan. The possible mechanism reaction of proteins as a coagent in peroxide cross-linked NR adapted from ref. [75] is given in Figure 4. When the radical is formed on the NR chain, the protein radical (a single protein molecule and/or an aggregate of cross-linked proteins) with more than one radical on that molecule is attached to the NR chain. Finally, the termination reaction can occur by the combination of radical intermediates, i.e., radicals on the NR and NR-proteins, leading to extra cross-links.

In addition, proteins can be oxidized by the presence of radicals and O_2_ to generate protein hydroperoxides [76,77,78,79] (more details and evidence of protein degradation will be discussed in the following sections). Although the concentration of O_2_ during the vulcanization process is quite low, some proteins might be oxidized by peroxide along with trace amounts of O_2_ to generate additional radicals in the vulcanization process.

NR samples always show higher cross-link density than DPNR because of the presence of proteins independently of the involved mechanism, which seems to be related to the higher concentration of radicals during the vulcanization process.

### 3.4. Evolution of Non-Elastic Network Defects During the Vulcanization Process

Even though most of the literature in the rubber field supposes perfect networks obtained from the cross-linking reaction to explain the elastic properties of those materials, non-elastic network defects, mainly dangling chain ends and loops, have recently been reported as an important factor to consider when describing the structure of vulcanized rubber samples [43,49] and understanding their stress–relaxation processes [80,81,82].

**Figure 5:**
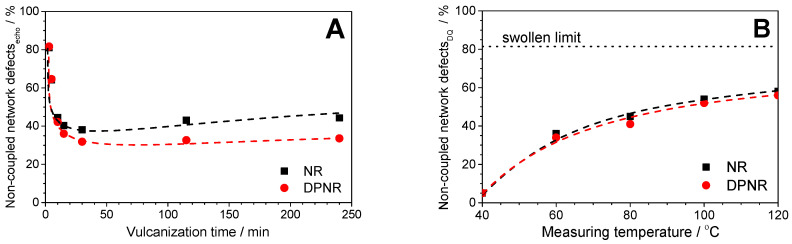
Evolution of non-coupled network defects for NR and DPNR as (**A**) a function of vulcanization time (measured at 40 °C in the swollen state by using Hahn echo experiments) and (**B**) as a function of the measuring temperature for samples vulcanized for 3 min (measured in the dry state by using DQ-NMR experiments). The swollen limit in (**B**) reflects the actual fraction of non-coupled defects for samples vulcanized for 3 min as measured by NMR in a swollen state at 40 °C from (**A**).

The non-elastic network defects for the NR and DPNR samples during peroxide vulcanization are shown in Figure 5A. Similar tendencies on the evolution of non-elastic network defects for the NR and DPNR up to the optimum vulcanization time (*t_95_*) were observed: the network defects decreased with the vulcanization time because of the formation of cross-links. At longer times, a slight increase in the fraction of network defects could be measured for the NR samples, whereas the DPNR seems to be a more stable sample with a constant number of defects above the optimum vulcanization times.

The first phenomenon is due to the progressive cross-linking reaction that reduces the fraction of un-cross-linked chains and shortens the molecular weight of the chain ends. Nevertheless, in any case, the actual fraction of non-coupled network defects for the optimum cross-linked NR samples reaches values of 30–40%. This means that peroxide vulcanization seems to be a quite ineffective process to form elastic cross-linked network structures in NR, whereas the side reactions that provide dangling chains (e.g., chain scission processes) seem to be favored in comparison with other elastomers [43].

Obviously, this scenario depends on temperature (see Figure 5B) and the observed timescale, minimizing the effect of network defects on the mechanical properties. The actual fraction of non-coupled defects (measurable only in a swollen state) is always underestimated when it is measured in dry samples (see Figure 5B) because chain packing or topological constraints promote some restrictions on the chain ends that provide some anisotropy in the movement to those entangled segments. Therefore, the fraction of non-elastic network defects measured by NMR decreases drastically at lower temperatures. The clearest example is given by the samples vulcanized for 3 min, whose actual fraction of defects is around 80% (as measured in the swollen state), but only 5% of those polymer segments behave as non-elastic defects at 40 °C in the dry state in the NMR timescale.

These statements support the assumption that cross-linking and chain scission reactions are competing and sequential reactions during the vulcanization process of NR with peroxides [83]. Before the optimum vulcanization time, t_95_, the overall process seems to be dominated by the cross-linking reaction: an increase in cross-link density and elastic torque and a decrease in the non-coupled network defects. However, the chain scission reaction seems to govern this process after achieving the network formation at t_95_ for the NR samples. It is important to remark that the increase in non-elastic network defects observed above the optimum vulcanization time for the NR sample (Figure 5A) has neither a visible effect on the average molecular weight between constraints nor on the elastic torque measured in the rheometer as demonstrated in Figure 3. This could be explained by the combined (and opposite) changes in the network structure caused by the presence of competing reactions, the cross-linking and the chain scission that modify the spatial distribution of cross-links, as will be discussed in the following sections.

By comparing the NR and DPNR samples, it is possible to establish that the proteins have a negative effect in this complex process since the NR showed a higher fraction of non-elastic network defects than the DPNR. In this sense, the higher concentration of radicals due to the presence of proteins increases the cross-link density. It enhances the chain scission reactions that occur during the NR vulcanization with peroxides, contributing to the higher fraction of defects of the NR. In addition, the proteins can form ineffective cross-links, i.e., proteins bound to a single rubber chain without forming any bridge with other chains that create dangling and pendant proteins grafted to NR chains. Proteins can raise the fraction of network defects in NR vulcanized with peroxides independently of the assumed mechanism involved in the process.

### 3.5. Evolution of Spatial Distribution of Cross-Links During the Vulcanization Process

The ^1^H DQ-NMR experiments not only allow the calculation of an average cross-link density, but also provide information on the spatial distribution of cross-links. Previous works have demonstrated that NR samples vulcanized with peroxide exhibited a broader distribution of cross-links than sulfur-based networks [43,57], and, in some cases (according to the peroxide concentration, vulcanization temperature, and type of elastomer), they contained highly cross-linked areas (inhomogeneities) termed as clusters [84], which were formed by the subsequent radical addition mechanism (similar to a polymerization reaction) to the double bond on the chain backbone [85,86,87].

**Figure 6:**
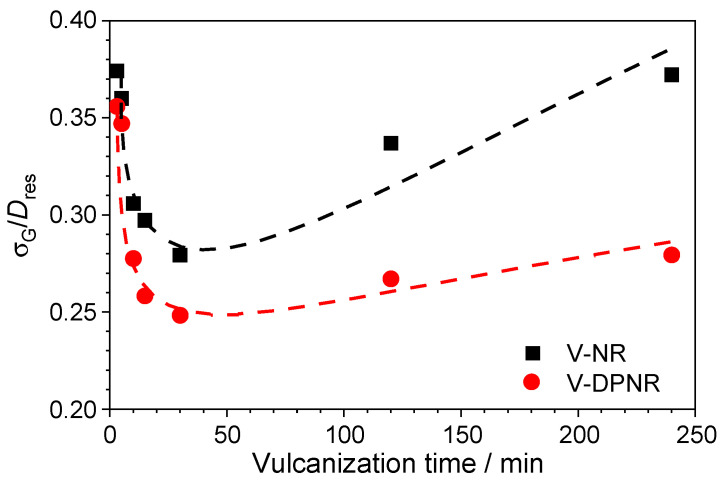
Variation in the relative width of the distribution of cross-link density as a function of vulcanization time for NR and DPNR, where σ represents the standard deviation and *D_res_* the average value of the dipolar coupling distribution.

The ratio between the standard deviation and the average value of the dipolar coupling distribution measured by NMR [47] is a robust parameter to describe a variation in the spatial distribution of the cross-links with the vulcanization time (see Figure 6). The quite broad cross-link distribution in the initial steps of vulcanization for both the NR and DPNR progressively decreased with the vulcanization time up to the optimum vulcanization time when it reached the narrowest distribution. At longer times (above the optimum vulcanization time), the scenario turned, and the distribution became broader again. This trend can be explained by the coexistence of two competitive reactions, i.e., the formation of cross-links and the chain scission reactions during the vulcanization process.

The spatial distribution of cross-links became narrower with the progress of vulcanization because the formation of additional cross-links randomly distributed (according to the radical pathway that governs the vulcanization based on peroxides) was dominant over other side reactions. Consequently, the cross-link density and spatial homogeneity of the formed network increased during this process.

**Figure 7:**
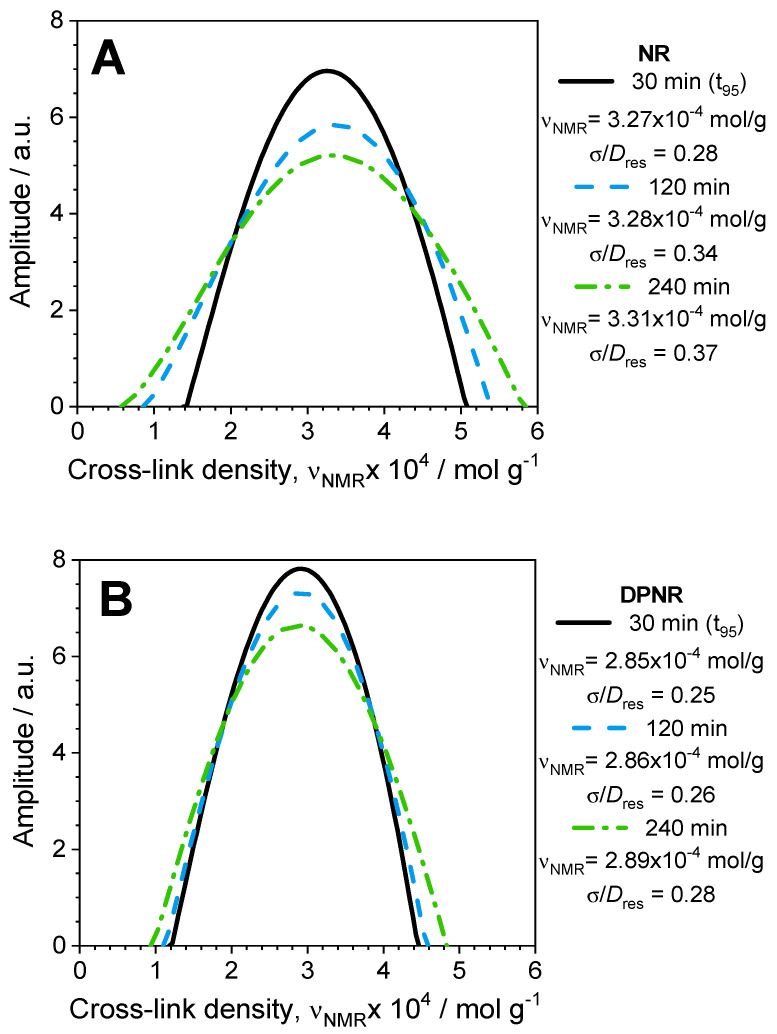
Spatial distribution of cross-links at various vulcanization times for (**A**) NR and (**B**) DPNR according to the regularization analysis of NMR data. Average cross-link density ($\upsilon_{NMR}$) and the relative width of the distributions ($\sigma /D_{res}$) are indicated.

To understand the network evolution over the optimum vulcanization time, it is mandatory to analyze in detail the actual spatial distribution of cross-links (see Figure 7). In the case of the NR, the distribution of cross-links became broader when the vulcanization time increased over *t_95_* (at 120 and 240 min, respectively) because both extremes in the distribution shifted to lower and higher cross-link densities, respectively. This is molecular evidence about the opposite effects of the two main reactions during the vulcanization process: (i) chain scission reactions that increase the fraction of non-elastic network defects (see Figure 5A) and the molecular weight between cross-links (*M*_c_), and (ii) the cross-linking reactions that promote the formation of areas with higher cross-link densities. Both opposite processes seem to compensate for their effects, and, consequently, the average cross-link density (measured as the average value of *D_res_* distribution) and the elastic torque measured in the rheometer remain constant, as demonstrated in Figure 3.

The deproteinized sample, DPNR, did not show any effect derived from chain scission reactions over the optimum vulcanization time (completely aligned with the almost constant value in the non-coupled network defects, as shown in Figure 5A). In that case, the broadening of the spatial cross-link distribution was caused by the formation of additional cross-links thanks to the persistence of radicals in the sample.

Since the proteins in the NR increased the concentration of radicals in the system and enhanced both the cross-linking and the chain scission reactions, the NR sample showed a higher fraction of network defects, higher cross-link density, and broader spatial distribution of cross-links than the DPNR sample. Chain scission reactions were minimized, and their negative effect on the network structure was almost negligible. These effects were amplified at vulcanization times over t_95_, where the proteins were active in this complex process.

Nevertheless, it is important to point out that in both cases, the low concentration of added peroxide (only 1 phr) cannot allow for the formation of highly cross-linked areas or clusters as was previously demonstrated in NR for samples with higher concentrations of peroxide [43]. This type of local inhomogeneity in the network structure for peroxide vulcanized samples should not be confused with the larger length-scale inhomogeneities caused by protein aggregates in both the un-cross-linked [87] and peroxide cross-linked [63] NR samples as was observed by atomic force microscopy (AFM) and SANS techniques, respectively. The latter is a larger-scale inhomogeneity that is undetectable by the more local NMR technique, and it has no influence on the actual structural information analyzed from the NMR experiments.

Consequently, the protein aggregation in the peroxide cross-linked NR needed to be confirmed, and this could be clarified by AFM and nitrogen analysis. The protein aggregations in the peroxide cross-linked NR were found even after leaching with hot water, which is an effective method for removing proteins from sulfur cross-linked NR products [88,89,90,91,92]. Furthermore, the nitrogen content of the peroxide cross-linked NR insignificantly changed after the leaching process (see Supplementary Information, Figure S3). These results signify that protein aggregates can be covalently bonded to the rubber backbone during the vulcanization of NR with peroxide, unlike the sulfur cross-linked NR.

It should be noted that NMR experiments are sensitive to very local information on the length scale of 10 nm, whereas AFM is measured in the micrometer scale. These techniques provide complementary information on different length scales, allowing us to conclude that the NR samples vulcanized with peroxide show inhomogeneities on different length scales caused by the consecutive addition reaction to the double bond (the formation of clusters of cross-links) and by the grafting of protein aggregates to the rubber chains.

**Figure 8:**
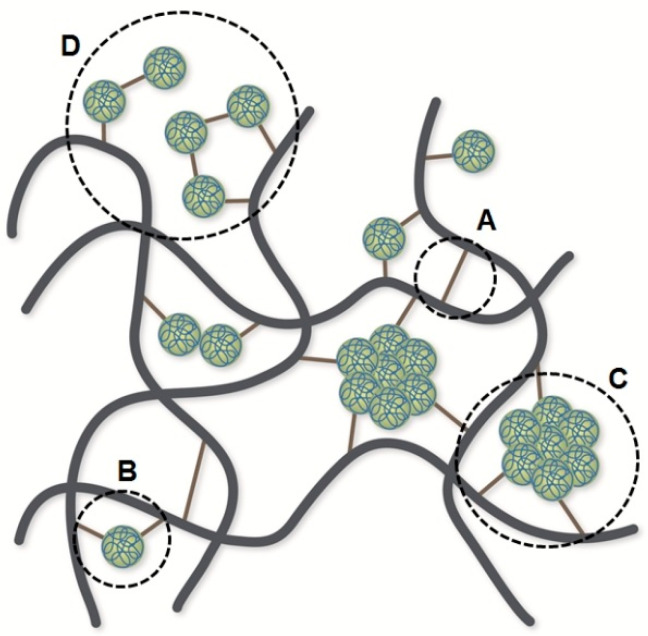
Schematic representation of the network structures associated with proteins in peroxide cross-linked NR. Cross-links are derived from (A) protein hydroperoxides, (B) the bridge of proteins, and (C) the aggregation of cross-linked proteins. No cross-link is derived from (D) dangling and pendant proteins grafted to the NR chain.

Based on the cross-link density, the fraction of non-elastic network defects, and the spatial cross-link distribution results obtained by NMR measurements and the AFM results in this work, the proposed network structures derived from the presence of proteins in peroxide cross-linked NR are schematically illustrated in Figure 8, where some additional cross-links are derived from protein hydroperoxides, the bridge of proteins, and the aggregation of cross-linked proteins according to AFM results, while the dangling and pendant proteins grafted to the NR chain do not form any cross-links.

### 3.6. Effect of Network Heterogeneities on the Mechanical Properties

The mechanical properties of vulcanized rubber depend on the different local parameters that define the network structure: the fraction of dangling chain ends; the cross-link density; the nature, functionality, and spatial distribution of cross-links; the effect of entanglements; and the junction mobility [82,93,94,95,96]. Several of these structural parameters are drastically modified during the vulcanization process, making it quite difficult to study the influence of each individual factor on the mechanical properties of rubber, which is usually dominated by the effect of cross-link density. Nevertheless, above the optimum vulcanization time, the variation in the cross-link density is almost neglectable for both the NR and DPNR samples, which provides an opportunity to evaluate the effect of other structural parameters in the tensile properties of those networks.

**Figure 9:**
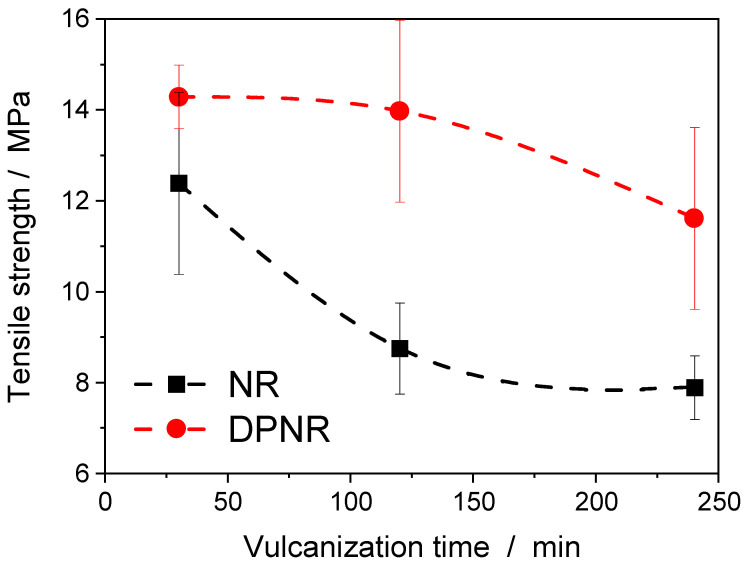
Variation in tensile strength with the vulcanization time for the NR and DPNR samples vulcanized with 1 phr of DCP at 160 °C.

Figure 9 shows that tensile strength decreases with the vulcanization time when the optimum time has been exceeded (t_95_ = 30 min) for the vulcanized NR and DPNR samples with 1 phr of DCP at 160 °C. The reported tensile strength values are the median of five test specimens, and the error bars represent their standard deviation. As discussed above, at those long vulcanization times, the coexistence of cross-linking reactions and chain scission processes in NR drives the formation of a higher fraction of non-elastic network defects and a broader spatial distribution of cross-links, but maintains the almost constant cross-link density in the sample. The absence of proteins reduces these negative reactions and the subsequent effects on the network structure of DPNR, showing higher tensile performance with lower cross-link density compared with NR.

**Figure 10:**
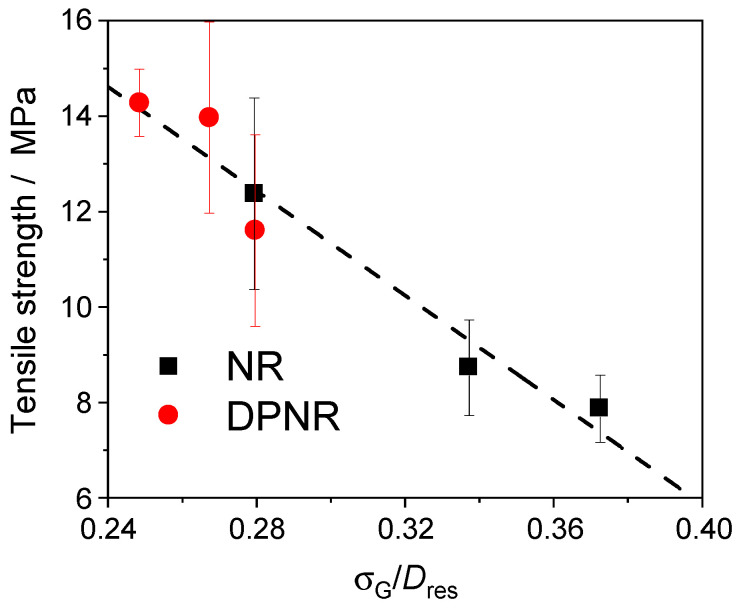
Relationship between tensile strength and the relative width of the spatial distribution of cross-links for the vulcanized rubber samples (NR and DPNR) with 1 phr of DCP at 160 °C.

On the one hand, Figure 10 shows a clear relationship between the decrease in the tensile strength and the relative width of the spatial distribution of cross-links, demonstrating that network inhomogeneities strongly influence the tensile strength of these rubber samples. On the other hand, the effect of non-elastic network defects on the tensile strength is difficult to evaluate. Although the presence of a high fraction of dangling chain ends (30–40%) was demonstrated in those samples, their elastic response depends on temperature and the observed timescale, minimizing the effect of these network defects on the mechanical properties.

These results suggest that rubber materials are not ideal networks because they contain non-elastic network defects (which should be considered) and a heterogeneous spatial distribution of cross-links. The latter seems to be a critical structural factor in understanding the ultimate tensile properties of rubber samples.

### 3.7. Thermal–Oxidative Degradation of Peroxide Cross-Linked NR

**Figure 11:**
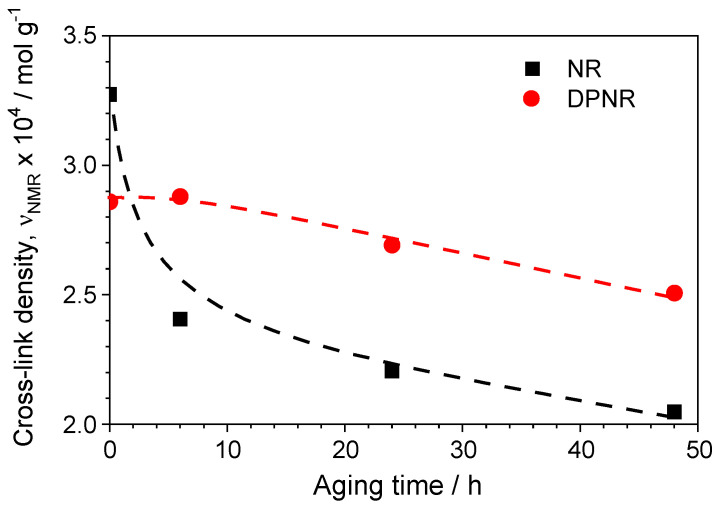
Evolution of cross-link density for the NR and DPNR samples (vulcanized at t_95_) as a function of aging time. Lines are a guide for the eyes.

The effects of some non-rubber constituents on the degradation behavior of NR have been reported. For example, tocotrienols exhibited antioxidant activity [97], whereas copper accelerated the degradation as a pro-oxidant [98]. The role of proteins has been also investigated on the degradation of NR, concluding that proteins showed either insignificant protection [99] or antioxidant activity [100] against thermal–oxidative aging. This might be due to a variety of testing conditions and analyzing techniques used for that purpose. To clarify this issue, the rubber samples were aged at 100 °C under air atmosphere for 6, 24, and 48 h in order to monitor the progressive degradation and evolution of the network structure by using NMR experiments. Figure 11 shows the changes in the cross-link density for NR and DPNR (vulcanized at their optimum vulcanization times, *t*_95_) at various aging times.

It was found that after 6 h of aging, the cross-link density for the NR remarkably decreased, while the DPNR sample showed no significant change with respect to the original structure. After that time, the cross-link density for both samples decreased with aging times up to 48 h in a similar way. These results evidence the active role of proteins in this process since they affect the degradation behavior of NR by increasing both the degradation rate and the final state of degradation. Nevertheless, it is important to remark that the effect of proteins seems to be limited to the initial stages of this thermo-oxidative process.

**Figure 12:**
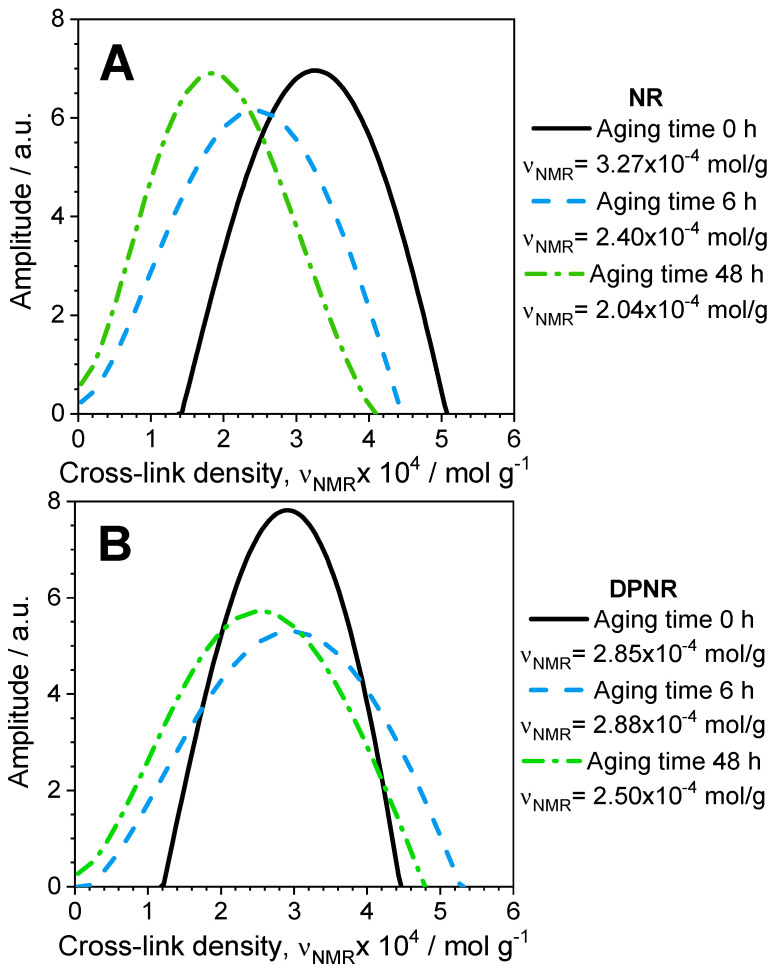
Spatial distribution of cross-link density at various aging times for (**A**) NR and (**B**) DPNR according to the regularization analysis.

Additional details about how networks are degraded were provided by the analysis of the actual spatial distribution of cross-links at various aging times, as shown in Figure 12. The degradation behavior for the DPNR and NR samples is, in both cases, complex. However, it is different for each sample: the spatial distribution of cross-links for the aged NR was only shifted towards lower cross-link densities by the preferential effect of the chain scission reaction (see Figure 12A), whereas the distribution for the DPNR sample was widened by both extremes, at lower and higher cross-link densities, respectively, due to the coexistence of the chain scission and cross-linking reactions during the degradation process (see Figure 12B).

Reactive oxygen species damage proteins at the protein backbones and the 20 common amino acid side chains, contributing to numerous protein degradation products such as carbonyl compounds, alcohols, and protein hydroperoxides [76,77,78,79]. The protein hydroperoxides can generate secondary radicals that induce damage to other proteins and other molecules as a chain reaction [78,101,102]. This is consistent with the assumption that proteins raise the rate and state of degradation in NR.

The analysis of the protein hydroperoxides of NR was carried out using extracted proteins as the model compound to confirm the formation of hydroperoxides caused by protein degradation (see Supplementary Information, Figure S4).

This confirmatory evidence indicates that the proteins in NR create hydroperoxides during the aging process, accelerating the degradation of other protein aggregates (further hydroperoxides can be generated as a chain reaction) and rubber chains. Consequently, the proteins in NR can prove to be pro-oxidant molecules that actively affect the degradation behavior of peroxide cross-linked NR and minimize the cross-linking reactions via radical mechanisms in the presence of oxygen.

## 4. Conclusions

Biopolymers are materials that can be derived from renewable resources or synthesized using a combination of renewable feedstocks and traditional polymerization methods, as observed in bio-based polymers. These biopolymers and bio-based polymers present a sustainable alternative to conventional petroleum-based polymers, being sourced from diverse feedstocks such as agricultural products (e.g., corn or soybeans) and non-traditional sources like algae or food waste. Additionally, these polymers are engineered with properties including biodegradability and composability.

The role of proteins on the pseudo-end-linked network, which characterizes the un-vulcanized NR, and their effects on the network formation and degradation of peroxide cross-linked NR was elucidated by time-domain ^1^H-NMR experiments. This experimental procedure has proven to be a successful approach to obtain quantitative and complete local information about the actual fraction of non-elastic network defects, the cross-link density, and its spatial distribution in NR without any interference from proteins and their aggregates. This is a superior advantage shown by NMR approaches with respect to qualitative swelling experiments and the small-angle neutron scattering (SANS) technique that is intensely disturbed by the strong scattering phenomenon of protein aggregates.

In un-vulcanized NR samples, the accessible conformational space for chain segments is always restricted with respect to the synthetic polyisoprene (IR) counterpart because of the presence of a pseudo-end-linked network as was demonstrated by ^1^H DQ-NMR experiments. The successive subtraction of proteins and phospholipids from the NR samples has demonstrated the main role of micelle formation or the association of the polar head-groups of phospholipid molecules at the α-terminal of the rubber chain as the cross-linking points of the naturally occurring networks, whereas the effect of proteins as cross-linkers at the NR chain ends seems to be minimal.

Although proteins have a negligible effect on the pseudo-end-linked network formed in un-vulcanized NR samples, they played an active role as coagents during the peroxide vulcanization of this material. It has been demonstrated that this type of vulcanization is a highly ineffective complex process for the NR matrix because at least two main competing reactions take place: cross-linking and chain scission phenomena. The existence of proteins in NR increases the concentration of radicals and provokes the effect on both opposite reactions. Consequently, the NR always showed a higher fraction of network defects, higher cross-link density, and broader (more heterogeneous) cross-link distribution than its deproteinized counterpart (DPNR). It is important to note that the extraction of proteins minimizes the importance of chain scission reactions, making their effects on the network structure negligible during the vulcanization process (in the absence of oxygen).

In addition to the local spatial inhomogeneities observed by NMR, NR samples vulcanized with peroxides also contain aggregates of proteins covalently grafted to the rubber backbone, as demonstrated by AFM. Those aggregates form larger length-scale inhomogeneities (in the range of microns) compared to the size of the highly cross-linked areas (in the range of tens of nm) in the rubber network structure. The latter has a deep impact on the tensile strength properties of these rubber samples.

Finally, proteins have a detrimental effect on the thermo-oxidative degradation of peroxide cross-linked NR because they act as pro-oxidant agents generating hydroperoxides that accelerate the degradation process. As a consequence, chain scission reactions are enlarged during this radical process to increase both the degradation rate and the final state of degradation. Contrary to this behavior, both opposite processes take place in deproteinized NR, and the spatial distribution of cross-links expands to both extremes, the higher and lower cross-link densities, although the chain scission reactions are predominant.

## Figures and Tables

**Table 1:** The characteristics of the rubber samples.

Samples	Protein Content (% *w*/*w*)	Ester Content (mmol/kg-Rubber)	Metal Content (% *w*/*w*)		
			Cu^2+^	Fe^2+^	Mn^2+^
NR	2.62	14.28	ND	0.0016	ND
DPNR	0.06	14.23	ND	0.0014	ND

## Data Availability

The original contributions presented in this study are included in the article/Supplementary Material. Further inquiries can be directed to the corresponding authors.

## Supplementary Materials

The following supporting information can be downloaded at: https://www.mdpi.com/article/10.3390/polym17081063/s1, Figure S1. Scheme of a proposed pseudo-end-linked network structure of NR (proteins at the ω-terminal and phospholipid molecules at the α-terminal). Figure S2. ESR spectrum and g value of the extracted proteins. Figure S3. AFM micrographs and nitrogen content for vulcanized NR, vulcanized DPNR and leached NR samples. The phase images were obtained by AFM tapping mode. Figure S4. Hydroperoxides content generated from extracted proteins at various aging times. Reference [103] is cited in the supplementary materials.
