# Supplementary material for: Effect of Proteins on the Network Formation and Degradation of Peroxide Cross-Linked Natural Rubber Elucidated by Time-Domain NMR
Source: Polymers (Basel). 2025 Apr 15;17(8):1063. doi: 10.3390/polym17081063 (PMC12030170; doi:10.3390/polym17081063)
Supplement: Supplementary file 1 [file polymers-17-01063-s001.zip › polymers-3473880-supplementary.pdf]

polymers

MDPI

Supplementary Information

Effect of Proteins on the Network Formation and Degradation of Peroxide Cross-Linked Natural Rubber Elucidated by Time-Domain NMR

Adun Nimpaiboon [1,2], Antonio González-Jiménez [3,4], Roberto Pérez-Aparicio [5], Fernando Martín-Salamanca [5], Zenen Zepeda-Rodríguez [5], Juan López-Valentín [5,*] and Jitladda Sakdapipanich [6,*]

[1] Rubber Technology Research Centre (RTEC), Faculty of Science, Mahidol University, Nakhon Pathom 73170, Thailand; adun.nim@mahidol.ac.th
[2] Department of Chemistry and Center of Excellence for Innovation in Chemistry, Faculty of Science, Mahidol University, Bangkok 10400, Thailand
[3] Plastic Research Center (AIMPLAS), València Parc Tecnològic, C/Gustave Eiffel 4, 46980 Paterna, Spain; angonzalez@aimplas.es
[4] Escuela Superior de Ingeniería y Tecnología (ESIT), Universidad Internacional de La Rioja (UNIR), Av. de la Paz 137, 26006 Logroño, Spain
[5] Institute of Polymer Science and Technology (CSIC), C/Juan de la Cierva 3, 2800 Madrid, Spain; rperezaparicio@gmail.com (R.P.-A.); fms@ictp.csic.es (F.M.-S.); zenen@ictp.csic.es (Z.Z.-R.)
[6] Department of Chemistry and Center of Excellence for Innovation in Chemistry, Faculty of Science, Mahidol University, Nakhon Pathom 73170, Thailand
* Correspondence: jlvalentin@ictp.csic.es (J.L.-V.); jitladda.sak@mahidol.ac.th (J.S.)

Academic Editor: Amitesh Maiti

Received: 26 January 2025
Revised: 14 March 2025
Accepted: 08 April 2025
Published: 15 April 2025

Citation: Nimpaiboon, A.; González-Jiménez, A.; Pérez-Aparicio, R.; Martín-Salamanca, F.; Zepeda-Rodríguez, Z.; López-Valentín, J.; Sakdapipanich, J. Effect of Proteins on the Network Formation and Degradation of Peroxide Cross-Linked Natural Rubber Elucidated by Time-Domain NMR. *Polymers* **2025**, *17*, 1063. https://doi.org/10.3390/polym17081063

Copyright: © 2025 by the authors. Licensee MDPI, Basel, Switzerland. This article is an open access article distributed under the terms and conditions of the Creative Commons Attribution (CC BY) license (https://creativecommons.org/licenses/by/4.0/).

I. Additional sample preparation

Transesterified deproteinized natural rubber (TE-DPNR)

Deproteinization of natural rubber (NR) was previously carried out as described in the main experimental section. Small pieces of the deproteinized NR (DPNR) were dissolved in toluene and then treated with freshly prepared sodium methoxide ($NaOCH_3$) under a nitrogen atmosphere at room temperature for 3 h. The resulting rubber solution was neutralized by methanolic-HCl and then subjected to centrifugation to remove any precipitates. The supernatant was purified by reprecipitation in methanol twice, followed by drying in a vacuum oven at 40°C for 24 h. The ester content of TE-DPNR measured by FTIR was 0 mmol/kg rubber.

Synthetic polyisoprene rubber (IR)

The commercial grade IR-2200 was produced by Zeon Corporation. In order to remove an insoluble component, the rubber was purified by dissolving at 0.15% w/v in toluene and then was centrifuged at 9,000 rpm for 45 min. The soluble fraction was precipitated in methanol and dried in a vacuum oven at 40°C for 24 h.

The extracted proteins of NR

Fresh NR latex was stabilized by 1% w/v sodium dodecyl sulfate and then kept in the dark at 4°C overnight, followed by centrifugation at 4°C for 60 min at the speed of 19000 rpm to obtain a clear serum phase. The serum phase was added with 3 times the volume of cold acetone. The mixture was kept at 4°C for 12 h in order to precipitate the proteins in the serum. The extracted proteins were collected and dried in a vacuum oven at room temperature for 12 h.

II. Additional characterization

Electron spin resonance (ESR) spectrometer

Radical species were detected by ESR spectroscopy using JEOL (model JES-RE2X) with microwave frequency at 9.6 GHz. ESR signal intensities were obtained by integration using ES-IPRIT software. The position of ESR absorption is expressed in terms of *g* value.

Atomic force microscopy (AFM)

Visualization of the rubber samples was conducted at room temperature using a NanoScope III an atomic force microscope from Digital Instruments (Santa Barbara, CA) equipped with a J scanner for a maximum scan area of 150 μm. The phase images of peroxide cross-linked NR films were recorded in a tapping mode using silicon tips (Nanosensors, Germany).

The quantity of protein hydroperoxides

The protein hydroperoxides generated from the extracted proteins of NR were detected by Pierce™ Quantitative Peroxide Assay Kits that measure hydrogen peroxide in protein samples based on the reaction of iron and xylenol orange reagents.

III. Additional results and discussion

The deproteinization process decomposes the network formed by protein components in the DPNR, wherein the network derived from phospholipids still remains. Additional transesterification treatment removes the phospholipids and avoids the formation of an end-linked network. The proposed pseudo end-linked network structure of NR, including the structural change after deproteinization and transesterification, can be seen in the Supplementary Information, Figure S1

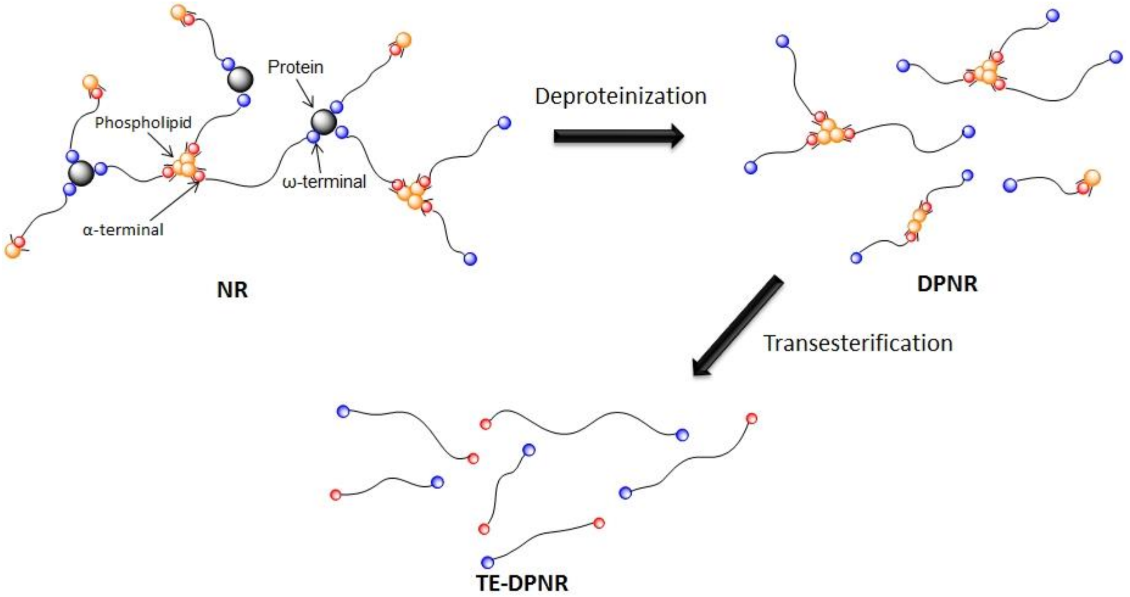

Figure S1. Scheme of a proposed pseudo-end-linked network structure of NR (proteins at the ω-terminal and phospholipid molecules at the α-terminal). Deproteinization decomposes proteins, while transesterification of DPNR decomposes phospholipids to liberate linear molecules [1].

The electron paramagnetic resonance (ESR) technique (Supplementary Information, Figure S2) can detect the presence of radicals on the protein of NR. The *g* value sited at 2.004 corresponds to the carbon radicals on the backbone and the side chain of proteins.

The phase image is used to detect variation in composition by ascribing based on the difference of energy dissipation, in which bright and dark phases represent hard and soft phases, respectively. From Figure S3, the phase image of the vulcanized NR revealed the bright phase, which is the harder phase in the matrix, whereas the darker and more homogeneous phase was observed in V-DPNR. Since proteins were mostly removed from vulcanized DPNR, as evidenced by the very low nitrogen content, the bright phase in NR should infer the protein aggregates. The vulcanized NR was leached with hot water (100°C) several times in order to remove proteins [2-6]. The bright phase as protein aggregates still remained, as can be seen in the phase image of leached NR.

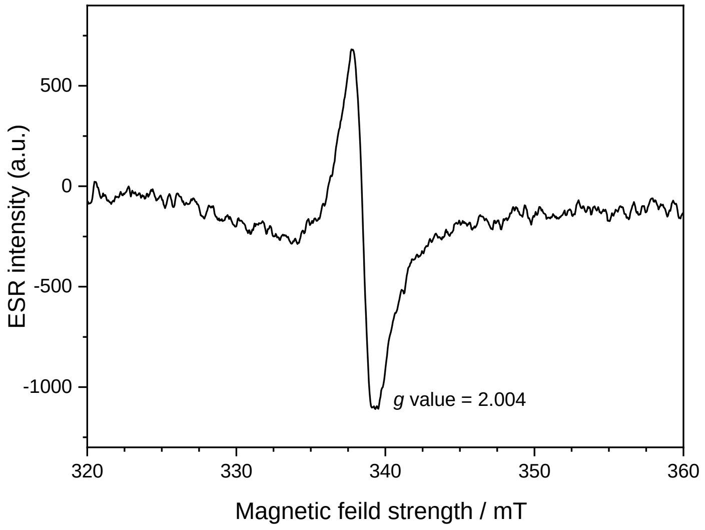

Figure S2. ESR spectrum and *g* value of the extracted proteins.

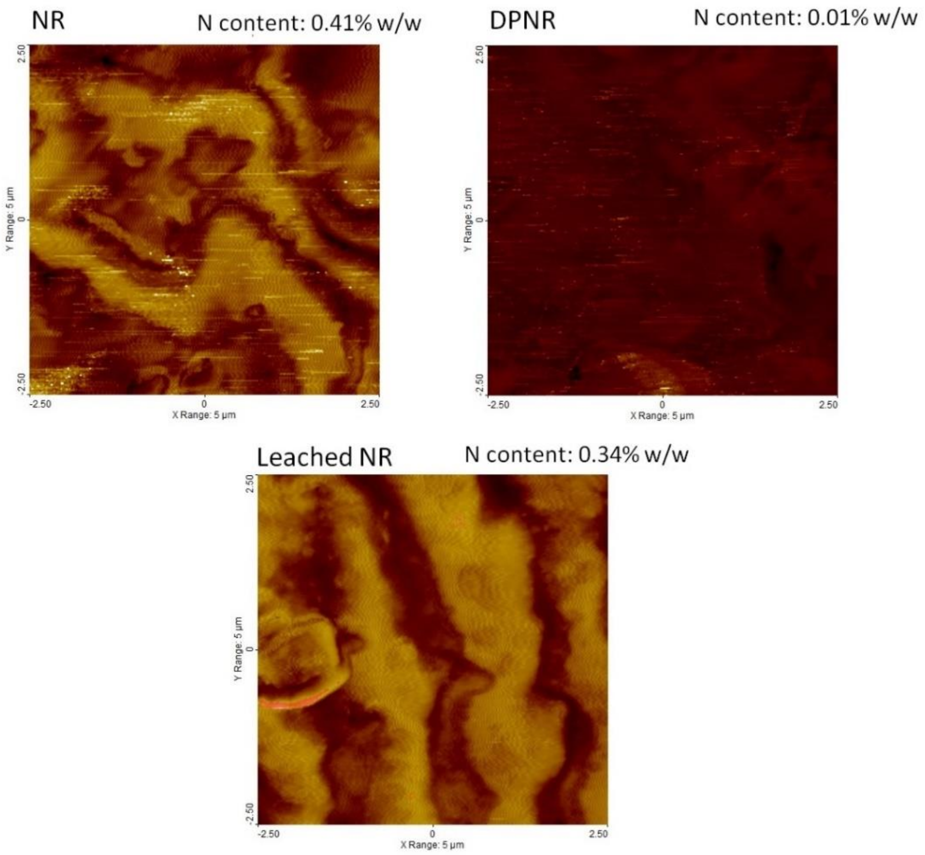

Figure S3. AFM micrographs and nitrogen content for vulcanized NR, vulcanized DPNR and leached NR samples. The phase images were obtained by AFM tapping mode.

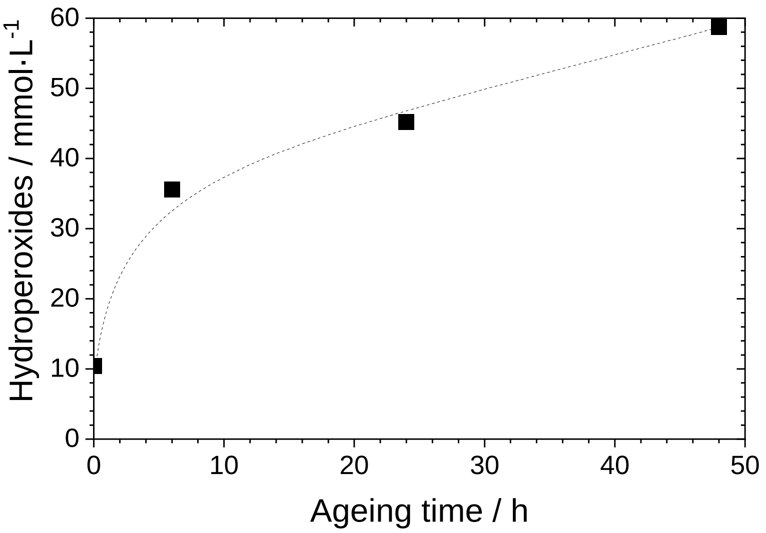

Figure S4. Hydroperoxides content generated from extracted proteins at various aging times.

The extracted proteins of NR were aged at 100°C under the same aging conditions as NR. Figure S4 demonstrates the occurrence of hydroperoxides and the increase in concentration of these species with the aging time. At this point, the given values could be underestimated with respect to the actual concentration of hydroperoxides during the NR

aging because the effect of residual DCP as additional sources of oxidative species during that process was not taken into consideration.

References

1. Tanaka Y, Tarachiwin L. Recent advances in structural characterization of natural rubber. Rubber Chem Technol 2009; 82:283-314.
2. Pailhories G. Reducing proteins in latex gloves: the industrial approach. Clin Rev Allergy 1993; 11:391-402.
3. Ng KP, Yip E, Mok KL. Production of natural rubber latex gloves with low extractable protein content: some practical recommendations. J Nat Rubber Res 1994; 9:87-95.
4. Ghazaly HM. Factory production of examination gloves from low protein latex. J Nat Rubber Res 1994:9:96-108.
5. Dalrymple SJ, Audley BG. Allergenic proteins in dipped products: factors influencing extractable protein levels. Rubber Dev 1992; 45:51-60.
6. Leynadier F, Tran Xuan T, Dry J. Allergenicity suppression in natural latex surgical gloves. Allergy 1991; 46:619-25.

Disclaimer/Publisher's Note: The statements, opinions and data contained in all publications are solely those of the individual author(s) and contributor(s) and not of MDPI and/or the editor(s). MDPI and/or the editor(s) disclaim responsibility for any injury to people or property resulting from any ideas, methods, instructions or products referred to in the content.
